# Supplementary material for: Frailty and risk of cardiovascular disease and mortality
Source: PLoS One. 2022 Sep 19;17(9):e0272527. doi: 10.1371/journal.pone.0272527 (PMC9484650; doi:10.1371/journal.pone.0272527)
Supplement: Attachment — Submitted filename: Responce to Reviewers.docx [file pone.0272527.s002.docx]

*Dear Reviewers,*

*Thank you very much for taking your time to review our manuscript and for providing us with valuable comments. Please see our reply below:*

Reviewers' comments:

Reviewer's Responses to Questions

**Comments to the Author**

1. If the authors have adequately addressed your comments raised in a previous round of review and you feel that this manuscript is now acceptable for publication, you may indicate that here to bypass the “Comments to the Author” section, enter your conflict of interest statement in the “Confidential to Editor” section, and submit your "Accept" recommendation.

Reviewer #1: All comments have been addressed

Reviewer #2: (No Response)

*Reply to Reviewer #2:*

*Thank you very much for all your comments. We’ve revised the manuscript according to your comments.*

2. Is the manuscript technically sound, and do the data support the conclusions?

The manuscript must describe a technically sound piece of scientific research with data that supports the conclusions. Experiments must have been conducted rigorously, with appropriate controls, replication, and sample sizes. The conclusions must be drawn appropriately based on the data presented.

Reviewer #1: Yes

Reviewer #2: Partly

*Reply to Reviewer #2:*

*We acknowledge that our manuscript may not be technically perfect. We have made every effort to minimize confounding bias in the data analysis. Our re-analysis of the data involves additional adjustment for potential confounding variables to make the interpretation of the results and conclusion reasonably sound.*

3. Has the statistical analysis been performed appropriately and rigorously?

Reviewer #1: Yes

Reviewer #2: N/A

4. Have the authors made all data underlying the findings in their manuscript fully available?

The [PLOS Data policy](https://ddec1-0-en-ctp.trendmicro.com/wis/clicktime/v1/query?url=http%3a%2f%2fwww.plosone.org%2fstatic%2fpolicies.action%23sharing&umid=e67acdb7-1f09-4e1f-80ef-4d3a6e79cce4&auth=8d3ccd473d52f326e51c0f75cb32c9541898e5d5-e4db967f856ff44f3bbe4d4659aa5270fe9a6b86) requires authors to make all data underlying the findings described in their manuscript fully available without restriction, with rare exception (please refer to the Data Availability Statement in the manuscript PDF file). The data should be provided as part of the manuscript or its supporting information, or deposited to a public repository. For example, in addition to summary statistics, the data points behind means, medians and variance measures should be available. If there are restrictions on publicly sharing data—e.g. participant privacy or use of data from a third party—those must be specified.

Reviewer #1: Yes

Reviewer #2: Yes

5. Is the manuscript presented in an intelligible fashion and written in standard English?

PLOS ONE does not copyedit accepted manuscripts, so the language in submitted articles must be clear, correct, and unambiguous. Any typographical or grammatical errors should be corrected at revision, so please note any specific errors here.

Reviewer #1: Yes

Reviewer #2: Yes

6. Review Comments to the Author

Please use the space provided to explain your answers to the questions above. You may also include additional comments for the author, including concerns about dual publication, research ethics, or publication ethics. (Please upload your review as an attachment if it exceeds 20,000 characters)

Reviewer #1: The authors have addressed all the comments from the previous review.
I vote to accept this manuscript as authors have made significant changes to the questions posed to them.

Reviewer #2: Thank you for replies to my previous comments. Almost of those have been appropriately addressed by the authors; however, there is an issue to be revised in the text.

Although the authors have described in the term of DISCUSSION as follows “Our study provides clues to the mechanistic and development relationship by showing....”, it’s better to revise as follows “Our study may provide clues to the mechanistic and development relationship by showing....”.

*Reply to Reviewer #2:*

*Thank you very much for this suggestion. We’ve revised the manuscript accordingly on Page 17:*

*“Our study* ***may*** *provide clues to the mechanistic and development relationship by showing....”*

7. PLOS authors have the option to publish the peer review history of their article ([what does this mean?](https://ddec1-0-en-ctp.trendmicro.com/wis/clicktime/v1/query?url=https%3a%2f%2fjournals.plos.org%2fplosone%2fs%2feditorial%2dand%2dpeer%2dreview%2dprocess%23loc%2dpeer%2dreview%2dhistory&umid=e67acdb7-1f09-4e1f-80ef-4d3a6e79cce4&auth=8d3ccd473d52f326e51c0f75cb32c9541898e5d5-4896629d912ebd7e80d63ba2dded32eef058d374)). If published, this will include your full peer review and any attached files.


If you choose “no”, your identity will remain anonymous but your review may still be made public.


**Do you want your identity to be public for this peer review?** For information about this choice, including consent withdrawal, please see our [Privacy Policy](https://ddec1-0-en-ctp.trendmicro.com/wis/clicktime/v1/query?url=https%3a%2f%2fwww.plos.org%2fprivacy%2dpolicy&umid=e67acdb7-1f09-4e1f-80ef-4d3a6e79cce4&auth=8d3ccd473d52f326e51c0f75cb32c9541898e5d5-ad30ed7b5e2817afd24adf5fd8cab5e2a2e908d8).

Reviewer #1: No

Reviewer #2: No
